# Is there an association between body image and self-rated oral health in a sample of Brazilian adolescents?

**DOI:** 10.1590/1807-3107bor-2025.vol39.055

**Published:** 2025-05-12

**Authors:** Auristhela Martinez ACEVEDO, Ana Leticia Andries e ARANTES, Lilian Maria de Moraes ANDRADE, Eliane Rodrigues de FARIA, Michele Pereira NETTO, Renata Maria Souza OLIVEIRA, Ana Paula Carlos CÂNDIDO, Isabel Cristina Gonçalves LEITE

**Affiliations:** (a) Universidade Federal de Juiz de Fora – UFJF, School of Medicine, Graduate Program in Collective Health, Juiz de Fora, MG, Brazil.; (b) Universidade Federal de Juiz de Fora – UFJF, School of Biological Sciences, Department of Nutrition, Juiz de Fora, MG, Brazil.; (c) Universidade Federal de Juiz de Fora – UFJF, School of Medicine, Department of Collective Health, Juiz de Fora, MG, Brazil.

**Keywords:** Adolescent, Self-Assessment, Oral Health, Body Image, Self-Concept

## Abstract

The aim of this study was to evaluate the association of body image perception and self-assessment of oral health with other associated factors in a sample of Brazilian adolescents. This cross-sectional study involved a total of 281 adolescents aged 14 to 19 years enrolled in 29 public schools from May 2021 to December 2023 in the urban area of Juiz de Fora, state of Minas Gerais, Brazil. The total enrollment of 9,502 participants in the first stage of the study () was considered for sample size calculation. The prevalence of negative self-assessment of oral health in the adolescent population was estimated at 18%, with a standard error of 1%, a 95% confidence interval, and a 20% loss to follow-up. Participants completed a questionnaire via the Google Forms platform, which gathered information on their socioeconomic status, self-perception of body image, self-assessment of oral health, and self-esteem. Bivariate analysis and logistic regression models were used to evaluate the associations between variables. Variables with p < 0.10 in the bivariate analysis were included in the logistic regression model. Variables with p < 0.05 were retained in the final model. The final logistic regression model revealed that adolescents with a higher socioeconomic status rated their oral health as excellent or good. Additionally, those who reported not living with their parents and had a low level of self-esteem had a poor oral health self-assessment. Adolescents’ perception of their body image was not associated with oral health self-assessment. On the other hand, socioeconomic factors, family structure, and self-esteem influenced adolescents’ oral health self-assessment.

## Introduction

Adolescence is defined by the World Health Organization as the stage of life encompassing individuals aged 10 to 19 years.^1^Adolescents engage in behaviors that are typical of this stage of life, which can either promote health protective or pose risks to their health and well-being. Their unhealthy choices and behaviors affect their well-being and result in negative consequences that persist throughout life, increasing mortality and morbidity.^2^

Self-assessment of oral health is an important indicator that encompasses both physical and emotional components, as well as aspects of well-being and life satisfaction.^3^Studies involving adolescents’ self-assessment of oral health highlight the importance of this indicator, given that oral health status may be perceived differently during adolescence.^4-8^Socioeconomic factors have been strongly associated with oral health self-assessment outcomes, demonstrating that a lower socioeconomic status leads to a negative oral health self-assessment.^9,10^Furthermore, family structure can influence oral health, demonstrating that the lack of a parent’s presence can have a negative effect on the oral health of adolescents and preadolescents.^11,12^

Evidence suggests that adolescent oral health and quality of life may be affected by psychological circumstances such as self-esteem and positive body image,^13^but few studies have focused on establishing a direct relationship between body image and self-rated oral health.^14,15^

On the other hand, there is evidence that self-esteem has a strong independent effect on self-rated oral health.^16^Adolescents with lower self-esteem showed behaviors that were less favorable to their oral health when compared to their peers with higher self-esteem.^17^Also, an individual’s mental health, including self-esteem, mood, quality of life, social life and satisfaction with health services, is influenced by their oral health status.^18,19^

Self-perceptions of oral health and body image can be associated once they are modified by various social, cultural, psychological, and individual factors, and some behaviors and feelings that arise at this stage of life can have implications for individuals’ future development. Therefore, the present study aims to evaluate the association of body image perception and self-assessment of oral health with other associated factors in a sample of Brazilian adolescents.

## Methods

This cross-sectional study is part of the second stage of the “Estilo de Vida na Adolescencência” (EVA) study (Adolescent Lifestyle study). The first stage included 835 adolescents aged 14 to 19 years enrolled in 29 of the 49 urban public schools in Juiz de For a, state of Minas Gerais, that met the target age range. Participants were from the final year of elementary school (9th year) or from one of the three years of high school. The study was conducted from May 2018 to May 2019. The research project was submitted to, and approved by, the research ethics committee of the Federal University of Juiz de Fora (process no. 41702920.1.0000.5147).

Adolescents aged 14 to 19 years, enrolled in urban public schools in Juiz de for a, Minas Gerais, were included in the study. Those older than 18 years signed an informed consent form for their participation, whereas parents or legal guardians signed an assent form for those younger than 18 years. There were no exclusion criteria. Further information on sample size and data collection methods in the first stage has been published in a previous article.^20^

The second stage was conducted from May 2021 to December 2023, with a data collection period of one year and seven months, due to delays caused by the COVID pandemic and school vacations. Four data collection methods were used: WhatsApp and e-mail; contact with the adolescents’ fathers, mothers, or legal guardians; phone calls to the students; and snowball sampling (contact with former participants and school coordinators to find additional respondents). The data were collected using an online questionnaire created on the Google Forms platform.

Sample size was calculated using Epi Info software (version 7.2.2.6; Centers for Disease Control and Prevention, USA), taking into consideration the total enrollment used for the first stage of the study (9,502 participants). The prevalence of negative self-assessment of oral health in the adolescent population was estimated at 18%^6^; with a standard error of 1%, a 95% confidence interval, and a 20% loss to follow-up. The final estimated sample size was 281 adolescents.

Sex, age, skin color, parents’ occupations and income, and the adolescents’ family structure were included in the questionnaire. An indicator based on asset ownership was used for economic classification.^21^

Body image perception was measured by the silhouette scale (figures of female and male silhouettes numbered 1 to 9), as proposed by Stunkard et al. in 1983, validated for the Brazilian population by Scaglusi.^22^ To verify body dissatisfaction, adolescents had to indicate the figure that best represented their current size and the figure they would like to have. When the variation between the real and ideal silhouette was equal to zero, adolescents were classified as satisfied; otherwise, they were considered to be dissatisfied.^23^

**Figure:**
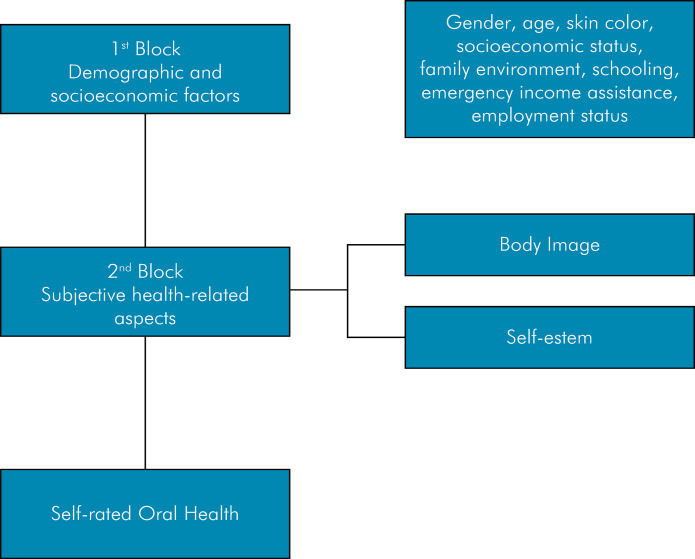
Theoretical model of variable hierarchization.

The self-esteem scale developed by Rosenberg^24^ (RSS) in 1989 was used to assess the level of self-esteem, and it was adapted, revised, and re-evaluated for Brazil by Hutz and Zanon^25^ in 2011. The items were answered on a four-point Likert scale ranging from 1- strongly agree, 2-agree, 3-disagree, and 4- strongly disagree.^25^By looking at the scores on this scale, the median RSS of the sample was used to classify the levels of self-esteem, a method used in previous research.^26,27^Thus, adolescents with scores less than or equal to 27 were classified as having low self-esteem.

Self-assessment of oral health (dependent variable) was assessed through a global self-assessment questionnaire: a) “Would you say that the health of your teeth, lips, jaws, and mouth is”: This variable was assessed on a five-point Likert scale, ranging from excellent (0) to poor (4).^28^For statistical analysis oral health was dichotomized as excellent/good and fair/poor according to Vettore et al.^8^ and Fagundes et al.^29^

A theoretical model was developed to guide the statistical analyses (Figure) and the study variables were dichotomized for the statistical analyses (Table 1). The classification of the sex variable was excluded from the bivariate and multivariate analyses (Table 2). The data were analyzed using SPSS software, version 21.0. Initially, a descriptive analysis of the data was performed (mean, frequencies, standard deviation, median, minimum, and maximum). Continuous variables were tested for normality using the Kolmogorov-Smirnov test, which indicated asymmetrical distribution. The crude odds ratio and respective confidence intervals allowed us to assess the association between the dependent variable and the independent variables. The chi-square test with Fisher correction was used whenever necessary. Variables with p < 0.10 in the bivariate analysis were included in the logistic regression model to control for significant variables between the hierarchical blocks. Variables with p < 0.05 were retained in the final model.

**Table 1:** Categorization of study variables of adolescents aged 14-19 years, enrolled in public schools in the urban area of Juiz de Fora, MG, 2021-2023.

Variable	Description /Data collection	Description for the analyses
Demographic and socioeconomic characteristics		
Sex	Male, Female, other	Male, Female
Age	-	14-17 years old, 18-29 years old
Race/Skin color	White, black, brown, yellow, indigenous, don’t know or don’t want to answer	White, other
ABEP	A, B1, C1, C2, D, E	A+B1+B2 and C1+C2+D+E
Adolescent living	Alone, only with father, only with mother, with father and mother, with grandfather and grandmother, with uncle and aunt, another	Father and mother/Father or mother, another
Resident under 18	Yes, No	Yes, No
	Illiterate/Elementary I incomplete, Elementary I complete / Elementary II incomplete, Elementary complete / High school incomplete, High school complete / University incomplete, University complete	
Father and mother working status	Homemaker, unemployed, formal job, informal job, retired or pensioner	Employee, Other
	No. Remained employed, No. Remained unemployed, Yes. Lost his/her job, Yes, got a job, Retiree or pensioner, Don’t know or don’t want to answer.	
Did your family receive emergency income assistance provided by the Government?	No. We did not apply for assistance, No. We were not approved, Yes, Don’t know or don’t want to answer.	Yes, No
Self-rated oral health		
Participation in household income		0-2 people, 3-9 people
“Would you say that the health of your teeth, lips, jaws and mouth is”	Excellent, very good, good, fair, poor	Excellent/good, Fair/poor
Body image		
Satisfaction with body image	Stunkard Figure Scale	Satisfied, Dissatisfied
Self-esteem		
Level of self-esteem	Rosenberg Self-Esteem Scale	High self-esteem >27, low self-esteem < 27

ABEP: Brazilian Economic Classification Criteria (Brazilian Market Research Association).

**Table 2:** Variables associated with negative self-assessment of oral health in adolescents aged 14–19 years enrolled in public schools in the urban area of Juiz de Fora, MG 2021–2023.

Variables	%	Raw OR (95%CI)	p-value	Adjusted OR (95%CI)	p-value
**Block 1 - Demographic and socioeconomic factors**					
Sex*			0.710		
Female	61.1	1.14(0.55–2.37)		–	-
Male	38.9	1		–	-
Age (years)			0.890		
14–16	51.2	0.94(0.40–2.18)		–	-
17–19	48.8	1		–	-
Race/Skin color			0.147		
White (a)	50.2	0.59(0.28–1.20)		–	-
Black / other	49.8	1		–	-
ABEP			0.001		0.039
A - B1 - B2	68.7	0.30(0.15–0.62)		0.70(0.51–0.98)	
C1 - C2 - D	31.3	1		1	
Adolescent living			0.011		0.082
Only with father / Only with	35.6	3.29(1.36–7.93)		0.98(0.40–2.38)	
Mother					
Other	18.9	3.48(1.29–9.41)		2.67(1.05–6.77)	
With father and mother	45.5	1		1	
Resident under 18			0.658		
Yes	63.3	1.18(0.56–2.47)		–	-
No	36.7	1		–	-
Father’s level of education			0.576		
Illiterate to incomplete high school	38.7	1.24(0.57–2.70)		–	-
High school complete to advanced degree	56.7	1		–	-
Mother’s level of education			0.943		
Illiterate to incomplete high school	37.7	0.97(0.46–2.03)		–	-
High school complete to advanced degree	62.3	1		–	-
Father’s working status			0.432		
Employed	74.1	1.45(0.56–3.7)	–	–	
Other	25.9	1		–	-
Mother’s working status			0.969		
Employed	68.3	1.01(0.47–2.18)		–	-
Other	31.7	1		–	-
Did your father’s working status change during the pandemic?		1.000			
No	85.0	0.96(0.31–2.98)		–	-
Yes	15.0	1		–	-
Did your mother’s working status change during the pandemic?		0.631			
No	73.2	0.44(0.19–1.00)		–	-
Yes	26.8	1		–	-
Did your family receive emergency income assistance provided by the Government?		0.279			
No	51.2	0.66(0.31–1.39)		–	-
Yes	48.8	1		–	-
Participation in household income			0.557		
0–2 people	70.8	0.80(0.37–1.68)		–	-
3–9 people		1			
**Block 2 - Variables of subjective health-related aspects**					
Body image			0.987		
Satisfied	33.5	1		–	
Dissatisfied	66.5	1.00(0.47–2.11)			
Level of self-esteem			0.004		
High self-esteem > 27	60.9	1		1	
Low self-esteem < 27	39.1	2.80(1.37–5.75)		2.98(1.41–6.29)	

ABEP: Brazilian Economic Classification Criteria (Brazilian Market Research Association); OR: Odds Ratio; CI: Confidence Intervals. * The subject described as other was excluded from the analysis.

## Results

Of the 835 adolescents selected from the first phase of the study, 610 remained, of which 225 were excluded (they were no longer within the age group), A total of 281 respondents were reached. Most participants were female (171 - 61.1%), with a mean age of 17.38 years (±1.23). Table 3 shows the characteristics of the sample according to the self-assessment of oral health. Thirty-six (12.8%) adolescents self-assessed their oral health as negative (fair/poor). As for body image, 187 (66.5%) adolescents were dissatisfied with it.

**Table 3:** Sample characteristics according to independent variables on self- assessment of oral health in adolescents aged 14–19 years, enrolled in public schools in the urban area of Juiz de Fora, MG 2021–2023.

Variables	Fair/poor		Excellent/good	
	n	%	n	%
**Block 1 - Demographic and socioeconomic factors**				
Sex				
Female	23	13.5	148	86.5
Male	13	11.9	96	88.1
Other	0	0.00	1	0.4
Age				
14	2	40.0	3	60.0
15	3	12.5	21	87.5
16	3	8.3	33	91.7
17	10	12.7	69	87.3
18	10	14.7	58	85.3
19	8	11.6	61	88.4
Race/Skin color				
White	14	9.9	127	90.1
Black	11	18.3	49	81.7
Brown	10	13.9	62	86.1
Yellow	0	0.0	2	100.0
Indigenous	0	0.0	1	0.4
Don’t know or don’t want to answer	1	20.0	4	80.0
ABEP				
A	2	4.8	40	95.2
B1	4	11.1	32	88.9
B2	10	8.7	105	91.3
C1	14	24.1	44	75.9
C2	5	20.0	20	80.0
D-E	1	20.0	4	80.0
Adolescent lives				
Alone	4	40.0	6	60.0
Only with father	1	9.1	10	90.9
Only with mother	17	19.1	72	80.9
With father and mother	8	6.2	120	93.8
With grandfather and grandmother	0	0.0	5	100.0
With uncle and aunt	1	33.3	2	66.7
Another	5	14.3	30	85.7
Resident under 18				
Yes	24	13.5	154	86.5
No	12	11.7	91	88.3
Father’s level of education				
Illiterate / Elementary I incomplete	2	8.3	22	91.7
Elementary I complete / Elementary II incomplete	7	18.4	31	81.6
Elementary complete / High school incomplete	4	12.5	28	87.5
High school complete / University incomplete	10	10.6	84	89.4
University complete	7	12.7	48	87.3
Don’t know or don’t want to answer	6	15.8	32	84.2
Mother’s level of education				
Illiterate / Elementary I incomplete	1	7.7	12	92.3
Elementary I complete / Elementary II incomplete	7	17.5	33	82.5
Elementary complete / High school incomplete	5	10.4	43	89.6
High school complete / University incomplete	13	13.4	84	86.6
University complete	9	12.9	61	87.1
Don’t know or don’t want to answer	1	7.7	12	92.3
What is your father’s employment status				
Homemaker	1	100.0	0	0.0
Unemployed	3	15.8	16	84.2
Formal job	19	14.3	114	85.7
Informal job	5	12.8	34	87.2
Retired or pensioner	2	5.0	38	95.0
Don’t know or don’t want to answer	6	12.2	43	87.8
What is your mother’s employment status				
Homemaker	6	13.6	38	86.4
Unemployed	2	9.1	20	90.9
Formal job	19	13.7	120	86.3
Informal job	5	11.4	39	88.6
Retired or pensioner	3	15.8	16	84.2
Don’t know or don’t want to answer	1	7.7	12	92.3
Did your father’s working status change during the pandemic?				
No. He remained employed	16	10.8	132	89.2
No. He remained unemployed	0	0.0	14	100.0
Yes. He lost his job	4	19.0	17	81.0
Yes, He got a job	4	28.6	10	71.4
He is a retiree or pensioner	2	5.4	35	94.6
Don’t know or don’t want to answer	10	21.3	37	78.7
Did your mother’s working status change during the pandemic?				
No. She remained employed	16	10.4	138	89.6
No. She remained unemployed	3	7.9	35	92.1
Yes. She lost her job	7	22.6	24	77.4
Yes, she got a job	3	20.0	12	80.0
She is a retiree or pensioner	4	21.1	15	78.9
Don’t know or don’t want to answer	3	12.5	21	87.5
Did your family receive emergency income assistance provided by the government?				
No. We did not apply for assistance	8	7.8	94	92.2
No. We were not approved	6	23.1	20	76.9
Yes	19	15.6	103	84.4
Don’t know or don’t want to answer	3	9.7	28	11.4
Participation in household income				
0–2 people	24	12.1	175	87.9
3–9 people	12	14.6	70	85.4
**Block 2 - variables of subjective health-related aspects**				
Body image				
Dissatisfied	24	66.7	163	87.2
Satisfied	12	12.8	82	87.2
Level of self-esteem				
Low self-esteem < 27	22	20.0	88	80.0
High self-esteem > 27	14	8.2	157	91.8

In terms of sociodemographic and economic factors, ABEP socioeconomic status (p < 0.001) as defined by the Brazilian Association of Population-based Studies (ABEP) and the family structure in which the adolescent lived (p < 0.01) were significantly associated (p < 0.05) with self-assessment of oral health . As for the subjective health-related variables, adolescents’ satisfaction with their body image had no significant association with self-rated oral health. Self-esteem was found to be associated with self-rated oral health (p < 0.004). The results in the bivariate analysis are shown in Table 2.

There was an association between body image satisfaction and level of self-esteem (OR: 1.71; 95%CI:1.01 2.89).

Adjusted odds ratios are presented in Table 2. Self-esteem maintained a significant association, as well as the socioeconomic status classified according to ABEP, with a protective effect of higher incomes. The variable related to family structure (whether adolescents lived with their parents) increased the frequency of negative self-assessment of oral health when adolescents reported not living with their parents.

## Discussion

No association was observed between body image and self-assessment of oral health. On the other hand, self-assessment of oral health was associated with socioeconomic status according to the Brazilian Economic Classification Criteria,^20^ type of family structure, and level of self-esteem. Adolescents who lived in families with a traditional structure and with a better household income were less likely to present a self-assessment of poor oral health, whereas those who had low self-esteem were more likely to present a self-assessment of poor oral health.

Few studies address the association between body satisfaction and self-rated oral health. A study conducted with male and female preadolescents in Romania found that those who were dissatisfied with their body image had a poor/regular self-assessment of oral health.^13^A study in New Zealand points out that adolescents with a high prevalence of dental caries and low self-esteem had higher body dissatisfaction.^14^Further studies should be conducted to confirm the hypothesis of association between these two variables, considering that adolescence is characterized by constant physical changes and beauty standards are shaped by sociocultural influences.^30^ Moreover, adolescents are influenced by the media, peers, and family members, resulting in an internalized concept of what an ideal body is. Additionally, such concept influences and is influenced by psychological factors, self-esteem, life satisfaction, and quality of life.^15,31^

Studies point out that adolescents express different perceptions about health and different lifestyles that may lead to lower self-care and fewer dental visits.^9,32^

Oral health is part of overall health status; therefore, it should be understood as an important part of individual development. In the present study, fair/poor self-assessment of oral health among adolescents had a prevalence of 12.8%. This percentage is lower than in other national surveys, in which the prevalence rates of fair/poor self-assessment of oral health in adolescents were 22.7%, 27.9%, and 39.3%. These studies have indicated that age, socioeconomic status, demographic characteristics, family structure, and clinical and psychological aspects are associated with self-assessment of oral health.^6,8,29^Among adolescents, those whose families had an income of more than five minimum wages were 30% less likely to have a negative self-assessment of oral health.

Results from the 2019 National Health Survey (NHS) and 2010 National Oral Health Survey (NOHS) point out that higher-income individuals had a better assessment of their general and oral health status. Adolescents living in an unfavorable socioeconomic environment have a greater impact in terms of pain/discomfort, functional limitations, and social impacts, and have more unequal access to dental services.^33^Socioeconomic status is a predictor of self-rated oral health and it also influences other oral health outcomes in a general context and reveals social inequalities in health.^10,29^

The family environment affects the health of individuals and is one of the main social determinants of health. The presence of both parents during adolescence appears to be important because the adoption of healthy habits and behaviors occurs at home. Parents influence the oral health of their children mainly through their own oral health-related behaviors, knowledge, and attitudes, and there is evidence that lack of support from fathers can influence adolescents’ negative self-assessment.^11,34^

Adolescents who lived with other people outside the traditional family structure were 2.96 times more likely to have a negative perception of oral health in the present study. These findings are in line with those of other studies, pointing out that adolescents or children who live within a traditional family structure, based on father and mother figures or with the presence of a parent, perceive their oral health as excellent/good.^11, 35^This allows us to infer that adolescents who fit into a normal family structure that is dictated by society will have the presence and support of both parents at home, so the influence of parents on education and health promotion will be more present in these adolescents, leading to good general health habits and perception.

Self-assessment of oral health is guided by socioeconomic factors and the family structure of individuals, and it is also influenced by psychosocial factors. Self-esteem has been shown to influence several dimensions of adolescents’ overall health, leading them to develop or adopt healthy or unhealthy behaviors in the short or long term.^34^

Self-esteem has been significantly related to adolescents’ dental esthetics, malocclusion, tooth loss, and untreated caries, which are strong predictors of low levels of self-esteem. Good self-rated oral health and perceived oral health-related quality of life, more frequent toothbrushing, and frequent use of oral health services are directly related to high self-esteem.^16,18,31^

The negative perception individuals have of their socioeconomic status can have a negative impact on their mental health, self-esteem, and internalized problems. This negative perception may be more evident in adolescence, as teenagers rely on social information to make judgments about themselves.^36^

Several studies emphasize that oral health self-assessment is linked to adolescents’ level of self-esteem and socioeconomic disparity. There are several hypotheses on whether the level of self-esteem influences oral health self-assessment or whether oral health self-assessment affects the level of self-esteem.^15^It was observed that low self-esteem is more likely to result in fair/poor self-assessment of oral health. Similarly, adolescents from low-income families seem to be more likely to have a low level of self-esteem, and these findings corroborate those of the present study.^37^

While sample size was achieved in this study and population characteristics were homogeneous, body satisfaction was not associated with self-assessment of oral health, and self-esteem was associated with both self-rated oral health and body satisfaction in adolescents. This latter association has been supported in the literature.^26,38^Body image of individuals is greatly impacted during and after puberty because of body changes , and self-esteem plays an important role in this equation.^39^

Given the cross-sectional design of this study, some limitations should be considered regarding causality. Moreover, as the study was conducted during the pandemic period and data were obtained online, it was difficult to reach the participants and, therefore, the respondents may present sociodemographic characteristics or a psychosocial profile that differs from that of the target population, making it difficult to generalize these data. On the other hand, the literature brings no systematic evidence of the association of self-assessment of oral health with body image, a subjective aspect particularly important for this population group, and no study involving the Brazilian population was identified.

## Conclusions

Adolescents’ perception of their body image was not associated with self-assessment of oral health; on the other hand, socioeconomic factors, family structure, and self-esteem influence adolescents’ self-assessment of oral health.

It is important that future studies evaluate how these factors impact the different processes experienced by adolescents and the possible consequences for the evaluation of oral health.
